# A Proposed Method for Accurate 3D Analysis of Cochlear Implant Migration Using Fusion of Cone Beam CT

**DOI:** 10.3389/fsurg.2016.00002

**Published:** 2016-01-25

**Authors:** Guido Dees, Marc van Hoof, Robert Stokroos

**Affiliations:** ^1^Department of Otorhinolaryngology and Head and Neck Surgery, Maastricht University Medical Center, Maastricht, Netherlands

**Keywords:** cochlear implant, migration, registration, cone beam CT

## Abstract

**Introduction:**

The goal of this investigation was to compare fusion of sequential cone beam computerized tomography (CT) volumes to the gold standard (fiducial registration) in order to be able to analyze clinical cochlear implant (CI) migration with high accuracy in three dimensions.

**Materials and methods:**

Paired cone beam CT volumes were performed on five human cadaver temporal bones and one human subject. These volumes were fused using 3D Slicer 4 and BRAINSFit software. Using a gold standard fiducial technique, the accuracy, robustness, and performance time of the fusion process were assessed.

**Results:**

This proposed fusion protocol achieves a subvoxel median Euclidean distance of 0.05 mm in human cadaver temporal bones and 0.16 mm (mean) when applied to the described *in vivo* human synthetic data set in over 95% of all fusions. Performance times are <2 min.

**Conclusion:**

Here, a new and validated method based on existing techniques is described, which could be used to accurately quantify migration of CI electrodes.

## Introduction

**Figure 1:**
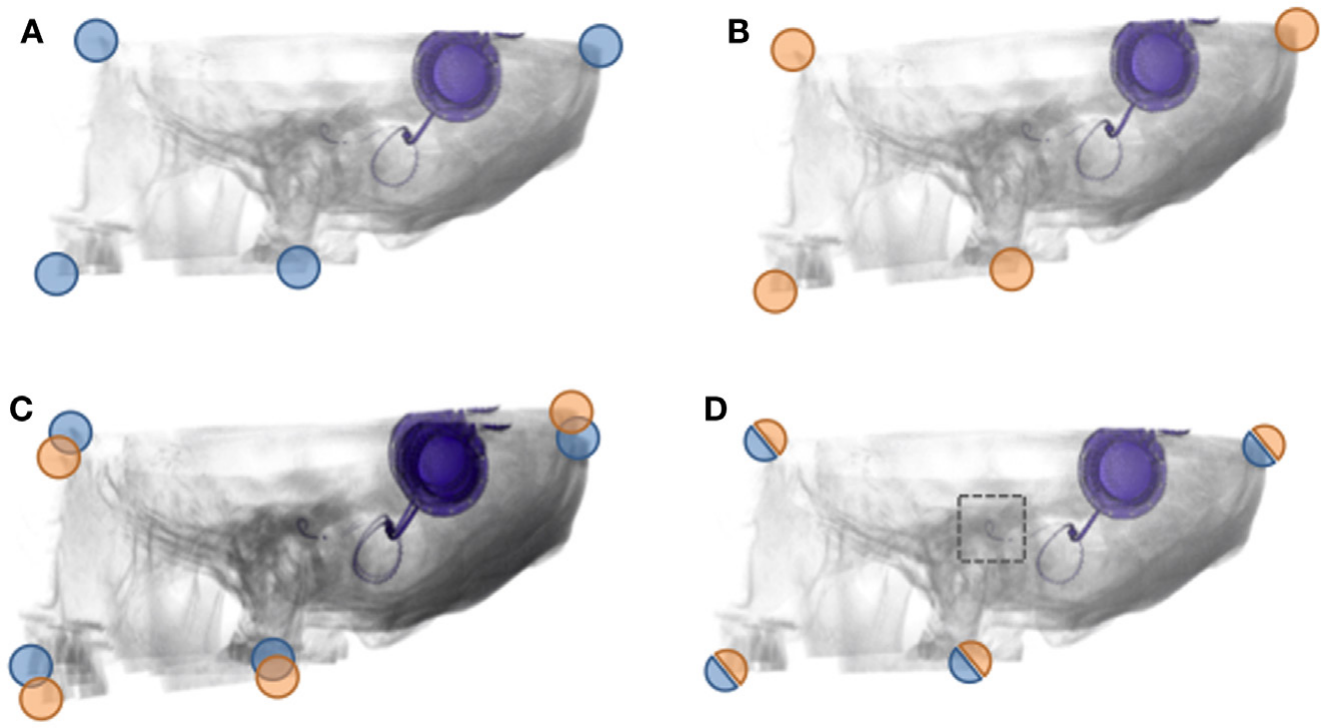
**Image fusion: registration and superimposition of CBCTs**. **(A,B)** An illustration of the first **(A)** and second CBCT **(B)**. The corner markings aim to represent the concept of fiducials (respectively, blue and orange). **(C)** An illustration of how CBCTs volumes fused without registration would look superimposed. Notice the discernible overlap, both in the image as the corner fiducials. **(D)** An illustration of how CBCTs volumes fused with registration would look superimposed. No discernible overlap is present, and the area of interest (dotted box) can be visually assessed. The fiducials are aligned as well.

**Figure 2:**
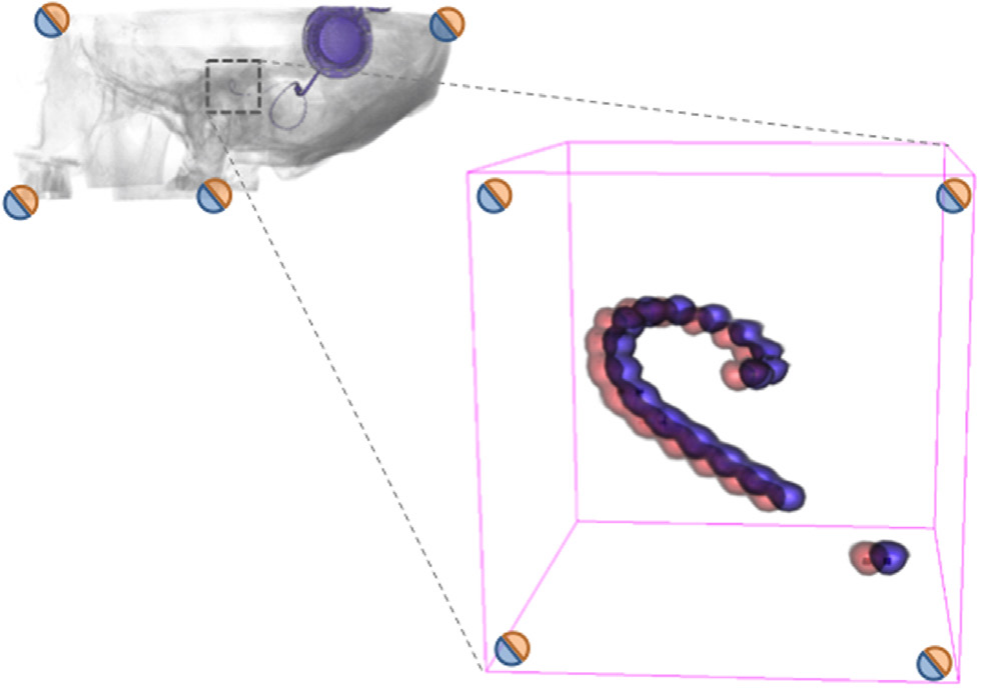
**Illustration of CI migration assessment**. When two volumes (CBCTs) are successfully fused using an accurate registration, the area of interest can be inspected for changes using volumetric rendering. In this exemplary imaginary case, there is a migration of the CI. Since the fiducials (corners) indicate the success of registration (as measured), one can establish the accuracy of the measurement of migration. The goal of the current investigation is to measure the distances between fiducials, so that it is possible to accurately determine the difference in CI electrode position, hence migration of the CI electrode.

The position of the cochlear implant (CI) electrode is of clinical importance (1). There have been reports of postoperative electrode movements, called migration. To detect a migration, one should know the CI’s initial position in the cochlea and have a follow-up measurement to determine if it remained in its initial position. Migration can occur in multiple directions of which the most important are in the direction of the cochlear duct (e.g., apically or basally) and toward or away of the modiolus. It can be expected that the pitch changes not only with the migration in the direction of the cochlear duct (2) but also with the distance to the modiolus (3). Moreover, the stability in the latter direction could hypothetically also be related to postoperative fibrosis (4). Thus, for an assessment of migration in all dimensions, one would need a method that is highly accurate in all three dimensions. Previously, a method has been applied, which can measure with an estimated accuracy of 1 mm in the direction of the cochlear duct (5). For determining the exact migration in every direction, a more accurate tool is needed. Here, a method is presented, which combines cone beam CT (CBCT) interval imaging with a freely availably software package. In essence, this software allows to fuse two CBCTs. The resulting difference can be assessed in three dimensions. Fusion of computerized tomography (CT) scans is possible using image registration, which aligns volumes in the same coordinate system (Figure 1). Stable, rigid parts depicted on an image, such as the temporal bone, should after fusion show no signs of overlap. If a CI migration would have occurred, this can be visually inspected, because there would exist a relative misalignment. This is illustrated by means of an example in Figure 2. The method introduced here relies on image fusion and is validated to determine how small the detectable migration in three dimensions can be.

## Materials and Methods

### Ethics

Ethical approval was sought from the medical ethics review board prior to our research involving the human subjects. This was deemed unrequired by the medical ethics review board in respect to our local legislations.

### Temporal Bones and the Human Subject

Two scans were performed on five temporal bones and one human subject. The second scan was performed directly after manually repositioning the temporal bones and altering the position of the human subject. An algorithm was applied to the second scan of the subject to create a realistic synthetic dataset of 200 scans. The algorithm applied random movements of the second scan within specific limits that mimic the variation in patient position in the CBCT. Additionally, a set of 200 volumes of interest (VOI) masks of 30 mm × 30 mm × 30 mm centered at the vestibular system was created using the same random movements.

### Cone Beam CT Examinations

Cone beam CT examinations were performed using a 17–19 I-CAT device (Imaging Sciences International, Hatfield, PA, USA) with a tube current of 37.07 mAs and a tube voltage of 120 kV. One full rotation took 26.9 s. Every scan results in a radiation exposure of approximately 0.05 mSv, based on in-house calculations. The raw data projection images were reconstructed using the I-CAT vision application (Imaging Sciences International) with an isotropic voxel size of 0.2 mm × 0.2 mm × 0.2 mm.

### Gold Standard and Software Analysis

An external skin fiducial marker containing three 0.3 mm Pb/Sn balls in diameter was developed. Three markers were applied on the temporal bone cadavers and four to register the scans of the human subject. The markers on the human subject were applied bilaterally on the mastoid. The mean of the Euclidean distances of the individual matched fiducials in two separate scans is used as an indication of fusion accuracy. A resulting Euclidean distance (from here on “distance”) <0.5 mm was deemed a successful fusion. The robustness of this method is expressed as a success percentage of the total fusions. Image analysis, registration, and visualization are performed using the open source 3D Slicer 4 package, including the BRAINS software package (6, 7) (available for download at www.slicer.org). The input parameters were based on the default parameters and information about its impact on performance and accuracy and subsequently optimized by an in-house developed algorithm. All custom algorithms, descriptive statistics, and Student’s *t*-test were executed in Wolfram Mathematica version 8.0 (Wolfram Research Inc., Champaign, IL, USA).

## Results

### Cadaver Temporal Bones

**Figure 3:**
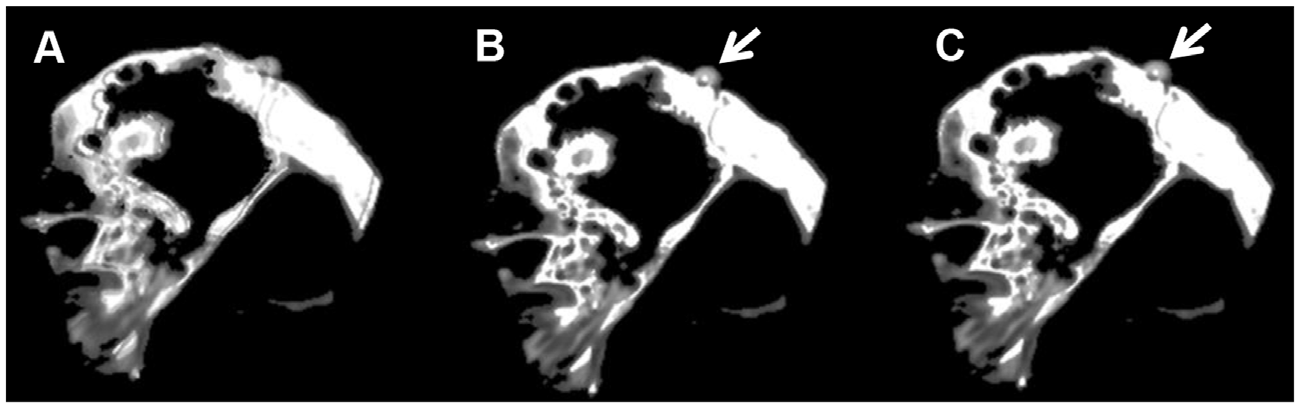
**Human cadaver temporal bone**. Axial fused images of a human cadaver temporal bone. **(A)** No registration. **(B)** Fiducial registration. **(C)** BRAINSFit registration. Notice the discernible overlapping borders when no registration is applied. The arrows indicate a fiducial marker.

The fiducial and BRAINSFit registered and superimposed (fused) volumes show no sign of overlap (Figures 3B,C) in comparison to the unregistered volumes (Figure 3A). The two non-registered volumes show a median distance of 4.1 mm. With fiducial registration, the median distance is 0.04 mm. Subsequently, the BRAINSFit registration shows a median distance of 0.05 mm (Table 1). The difference between the fiducial and BRAINSFit registration was not significant at 0.01 mm (*p* > 0.05). The average performance time to process the protocol was 44 s.

**Table 1:** **Temporal bone registration accuracy for different methods**.

Registration type	None			Fiducial			BRAINSFit		
Temporal bone	Mean	Min	Max	Mean	Min	Max	Mean	Min	Max
1	3.51	2.59	5.34	0.04	0.02	0.06	0.05	0.01	0.07
2	5.12	3.82	5.94	0.05	0.02	0.10	0.07	0.01	0.14
3	4.11	2.33	5.99	0.03	0.01	0.07	0.05	0.02	0.07
4	5.65	2.09	9.77	0.05	0.02	0.11	0.07	0.02	0.10
5	2.09	0.65	3.04	0.03	0.02	0.05	0.04	0.01	0.07
Median values	4.11	2.33	5.94	0.04	0.02	0.07	0.05	0.01	0.07

*The mean Euclidean distances before registration and after fiducial and BRAINSFit registration of fiducials on the five temporal bones. All values are given in millimeters*.

### *In Vivo* and *In Silico* Human Data Set

**Figure 4:**
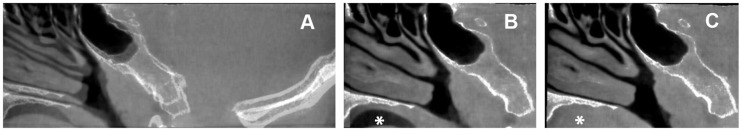
**Sagittal coupes of the head of the human subject**. Sagittal fused images of the human subject. **(A)** No registration. **(B,C)** Fiducial registration with the translucency, respectively, on the first and second volume. Asterisk indicates the different swallowing phase.

The evident initial volume alignment mismatch *in vivo* is visible at the transition between soft tissue and osseous tissues (Figure 4A). A different soft tissue configuration is introduced by swallowing during CBCT scanning by the subject (Figures 4B,C). Initially, without registration and fusion, a mean distance of 5 mm is found. Following fiducial registration and fusion, the mean distance is 0.06 mm. The mean distance is 0.16 mm for successful BRAINSFit registration with a maximum distance of 0.32 mm. When applying just the first step, a successful fusion in 95.5% is achieved. After a second step where BRAINSFit is set to use the second initialization alignment, the fusions are 100% successful. The difference between fiducial and BRAINSFit fusion was statistically significant at 0.1 mm (*p* < 0.01). The average performance time per registration was 95 s. The results when fusion is performed using a VOI are on average 0.1 mm larger (*p* < 0.01) than when registering using the total volume (mean 0.25 mm) with an initial success percentage of 100.

## Discussion

### Interpretation of the Results

In a less complicated environment, e.g., human cadaver temporal bone, there is no significant difference between fiducial registration and the BRAINSFit registration algorithm for achieving image fusion. However, in a realistic *in vivo* setting, the gold standard performs significantly better than the algorithm because of movement artifacts. Movements were present between CBCTs in the described results of the human subject and an example of this is shown by the different soft tissue configurations introduced by swallowing during scanning (Figures 3B,C). This does not implicate that the algorithm used here is deemed unfit for application in clinical practice. Application of fiducial registration for fusion is not feasible in the daily clinical routine. Indeed, actual patients cannot be retrospectively equipped with fiducial markers. Moreover, the accuracy of the algorithm in an *in vivo* environment is more than sufficient to determine any clinical relevant migration.

Surgical interventions and pathology modify anatomy over time. Hence, they could also affect the outcome of registration and fusion. The CI itself and its scattering and beam hardening artifacts can easily be filtered by excluding the 5% highest intensities present in both scans. However, the fusion of 200 synthetic volumes using a VOI demonstrates that, by selecting a stable VOI in an unaffected region, accurate (mean distance 0.25 mm) and robust fusion (success rate of 100%) is still achieved. In the rare case, the fusion still fails, one could convert to a semiautomatic method, using an approximate initial manual alignment. These results are comparable to adjacent surface- and intensity-based methods, as described in several other studies (8, 9). The average times needed to perform registration were favorable in contrast to surface-based registration, which has been reported to take too long for practical use (10). The time period for manual registration is reported to need 13 min (11) and fiducial- or landmark-based registration requires even more time (12).

### Clinical Applications and Future Perspectives

The method described in this article allows any clinician to verify visually if CI migration exists at any time postimplantation while meeting the prerequisites mentioned above. The accuracy of the method described (0.16 mm) would be sufficient to detect movement of a single electrode contact in three dimensions. Although CBCT was used in this article, we expect a similar performance when using high-resolution CT scanning and the proposed protocol. When one or multiple postoperative CT are fused with a preoperative MRI, one could possibly even assess how electrode contacts are positioned or migrated in relation to the inner-cochlear structures. This has been the goal for a prospective clinical study in CI users where the data collection has been finalized (*clinicaltrials.gov ID: NCT01940783*). Other interesting new applications of image fusion specific to ENT could include the follow-up of tumors, especially vestibular schwannomas.

### Limitations

Seemingly contradictory, no CI recipient was used nor was a CI inserted in the cochlea. The CI could have moved in the temporal bone while repositioning, negatively affecting the gold standard and accuracy measurements. Moreover, the CI represents only a fraction of the intensities (in Hounsfield units) on a scan, and they can be easily filtered by the software analysis. The advantages of the CBCT imaging modality come with the drawback of a relatively long scanning time. This could introduce imaging artifacts by movement of the subject.

## Conclusion

Here, a new and validated method is described, which could be used to accurately quantify migration of CI electrodes in three dimensions using image fusion.

## Author Contributions

GD: principal investigator, performed research described in this paper, and wrote this paper together with MH. MH: principal investigator, performed research described in this paper, and wrote this paper together with GD. RS: reviewed this paper as head of the neurotology division of the department.

## Conflict of Interest Statement

The authors declare that the research was conducted in the absence of any commercial or financial relationships that could be construed as a potential conflict of interest.
